# Peptide-based vaccine for cancer therapies

**DOI:** 10.3389/fimmu.2023.1210044

**Published:** 2023-08-16

**Authors:** Luigi Buonaguro, Maria Tagliamonte

**Affiliations:** Innovative Immunological Models Unit, Istituto Nazionale Tumori - IRCCS - “Fond G. Pascale”, Naples, Italy

**Keywords:** peptides, TAA, TME, molecular mimicry, immunopeptidome, combinatorial strategies, adjuvant, MHC

## Abstract

Different strategies based on peptides are available for cancer treatment, in particular to counter-act the progression of tumor growth and disease relapse. In the last decade, in the context of therapeutic strategies against cancer, peptide-based vaccines have been evaluated in different tumor models. The peptides selected for cancer vaccine development can be classified in two main type: tumor-associated antigens (TAAs) and tumor-specific antigens (TSAs), which are captured, internalized, processed and presented by antigen-presenting cells (APCs) to cell-mediated immunity. Peptides loaded onto MHC class I are recognized by a specific TCR of CD8+ T cells, which are activated to exert their cytotoxic activity against tumor cells presenting the same peptide-MHC-I complex. This process is defined as active immunotherapy as the host’s immune system is either *de novo* activated or restimulated to mount an effective, tumor-specific immune reaction that may ultimately lead to tu-mor regression. However, while the preclinical data have frequently shown encouraging results, therapeutic cancer vaccines clinical trials, including those based on peptides have not provided satisfactory data to date. The limited efficacy of peptide-based cancer vaccines is the consequence of several factors, including the identification of specific target tumor antigens, the limited immunogenicity of peptides and the highly immunosuppressive tumor microenvironment (TME). An effective cancer vaccine can be developed only by addressing all such different aspects. The present review describes the state of the art for each of such factors.

## 1 Introduction

In 1991, van der Bruggen et al, Ludwig’s researchers in Brussels, published in Science a groundbreaking study on tumor antigens. They described for the first time that cytotoxic T cells can selectively recognize a tumor antigen expressed by the human melanoma (MAGE Ag) (1). This article represents a pillar oftumor immunology introducing the concept that such specific antigens may be used to design and develop an effective active immunotherapy. Since the discovery of MAGE, the field has moved forward rapidly. Scientists have discovered many other tumor antigens, of which many are being tested as targets for immunotherapy and in particular for therapeutic peptide-based cancer vaccines (2, 3).

Active immunotherapy is aimed either at amplifying the existing antitumor immune response by nonspecific proinflammatory molecules/adjuvants or at eliciting a specific *de novo* host immune response against selected tumor antigens by cancer vaccines. In particular, peptide-based cancer vaccines are able to activate the effector adaptive immune response as well as to provide long-term acquired immunity against a “foreign” tumor antigen. Indeed, cancer cells can be distinguished from normal cells by either upregulation/overexpression of endogenous proteins or mutation of those proteins. As a result, any mutated or differentially expressed protein in cancer cells can potentially represent a vaccine target. In particular, the antigens derived from overexpressed self proteins in tumor cells are defined tumor-associated antigens (TAAs) and might be shared among patients with the same tumor (4) (5, 6). To this class of antigens belong cell lineage differentiation antigens, which are normally not expressed in adult tissue (7, 8); and cancer/germline antigens (also known as cancer/testis) (9). Since TAAs are also expressed by normal cells, they may be subject to immunological tolerance and therefore may be poorly immunogenic. In contrast, antigens arising from cancer-related nonsynonymous mutations or other genetic alterations in cancer cells, named tumor-specific antigens (TSAs), are not expressed on the surface of normal cells and are specific to each type of tumors (10–12). Consequently, TSAs are not subject to central and peripheral immune tolerance and are able to trigger a specific and effective T cell response against cancer cells (13).

The transformed cells are recognized by the immune system through immunosurveillance. Indeed, new antigens, including tumor antigens, are constantly presented by antigen-presenting cells (APC) to B and T lymphocytes of the adaptive immune response. The APCs can express the peptides through the MHC class I molecule for presentation to CD8+ T cells, or MHC class II molecule for presentation to CD4+ T helper cells. The latter may differentiate in two major subtypes, Th2 and Th1, involved in inflammatory response as well as in potentiating and sustaining the activity of CD8+ T cells (CTLs), respectively (14). Consequently, activated CD8+ T cells recognize cancer antigens expressed on the surface of tumor cells and initiate the release of apoptotic factors such as Perforin, Fas Ligand and Granzymes, leading to cell-mediated cytotoxicity (15). In this regard, the basic principles for a successful therapeutic vaccination against tumors include delivery of large amounts of high immunogenic antigens to APCs, induction of strong and sustained CD4+ T helper cell and CD8+ cytotoxic T lymphocyte (CTL) responses, infiltration of the TME as well as durability and maintenance of the immune response. In particular, strategies for improving activation and maturation of APCs, aiming at a more efficient antigen presentation to elicit an optimal T cell response, are actively pursued by several groups. Such strategies are mostly focused on the identification, selection and validation of novel adjuvants able to stimulate and enhance the magnitude and durability of antigen-specific T and B cell responses (16).Several therapeutic peptide-based vaccine strategies and formulations have been evaluated in different tumor types in the last 2 decades. However, only modest effects have been reported with an overall rate of clinical benefit of around 20% (17, 18). MUC1 represents a paradigmatic example of a TAA, highly expressed by adenocarcinomas, which has been evaluated in several human clinical trials with poor efficacy. In particular, MUC1 was targeted to dendritic cells, demonstrating the induction of highly specific immune response in preclinical and in human clinical trials (19). Subsequently, the ligand mannan used to target antigens in the mannose receptor lead to maturation of dendritic cells and activation via the Toll-Like Receptor 4 (20–24). The National Cancer Institute Translational Research Working Group has listed MUC1 as the second promising target in cancer research from 75 tumor-related antigens (25). The distinctive biological structure of MUC1 and its aberrant glycosylation in cancer cells make it a recognized tumor-specific antigen on epithelial tumor cells (26, 27). In particular, MUC1 consists of both a C-terminal fragment (MUC1-C), highly conserved region, and an N-terminal fragment (MUC1-N), which contains the variable number tandem repeat (VNTR) region. The latter has been described to play a critical role in the MUC1 immunogenicity.

Therapeutic MUC1−based vaccines have been tested in numerous early stage clinical trials (28); ClinicalTrials.gov https://clinicaltrials.gov/ct2/show/NCT02134925, but none of them has shown the expected anti-tumor effect in patients. In particular, these studies indicate that both the VNTR (MUC1-N) and non-VNTR (MUC1-C) regions contribute to immune evasion by cancer cells, which needs to be taken into consideration and addressed during the further development of MUC1-based cancer vaccines (29–31).

To date, the only FDA-approved therapeutic cancer vaccine is Provenge^®^ (Sipucleucel-T) for patients with castration-resistant prostate cancer (32). However, a modest increase in overall survival is observed together with a partial tumor regression. In addition to Sipucleucel-T vaccine, other peptide-based cancer vaccines are developed for prostate cancer, such as Cancer-associated membrane carbohydrates, including ganglioside (GM2), mucin 1 (MUC1), globo H and Thompson–Friedenreich antigen. Overall, five approaches - GVAX, DCVAC/PCa, a multi-epitope peptide vaccine, sipuleucel-T and PROSTVAC (33) - have been investigated in randomized phase III trials. They all showed safety and partial immunological activity but no effective clinical efficacy (34).

Such a poor efficacy of the therapeutic cancer vaccines developed for prostate cancer is common to most of the vaccines developed for other cancers. This is the consequence of a series of events that facilitate tumor development. Among these, the immunosuppressive TME plays a central role in cancer cell immune escape, inhibiting the activation of a specific T cell immune response against tumor cells (35). Moreover, during the tumor growth, the constant pressure from the adaptive immune system, coupled with the genetic instability of cancer cells, can select cellular sub-clones with reduced immunogenicity able to evade the immune recognition and destruction. Such low immunogenic sub-clones are often affected by loss of antigen presentation, due to a defective antigen- presenting machinery (36), hampering the recognition of tumor antigens by conventional (CD4+ and CD8+) and unconventional T cells (37). Moreover, chronically stimulated CD8+ T cells infiltrating the TME may acquire an ‘exhausted’ state characterized by loss of cytolytic activity, reduced cytokine production and reduced proliferation capacity.

To circumvent such adverse components of TME and improve their efficacy, cancer vaccines should be combined with immunomodulatory drugs (namely immunocheckpoint inhibitors, ICIs) to counterbalance the intra-tumor immune suppressive factors (38–40).

In the present review, we provide an overview of biological mechanisms underlying peptide-based vaccine platform developed in the last years for cancer therapy. We also describe the identification, characterization and design of such antigens, their formulation, as well as different combinatorial strategies to optimize their antigenicity and overcome the immune evasion for more specific and effective immune response.

## 2 Peptides for cancer vaccine development

### 2.1 Peptide MHC interaction

The immunodominance is a key aspect to consider when planning a vaccination strategy, in particular when the vaccine formulation is based on few and short specific epitopes. In particular, the immunodominance of T cell determinants results from several factors: 1) intrinsic characteristics of the epitope, e.g. the binding affinity to MHC molecules; 2) the presence of appropriate MHC molecules and T cell receptors at the individual level; and 3) competition between different epitopes of a given protein antigen for the available MHC binding sites. The combination of all such factors will give rise to distinct immunodominant epitopes selectively driving the T cell response.

Thus, to maximize the efficacy of a vaccine based on a mix of peptides, it is relevant to ensure a balanced T cell response for all the peptides included in the mix. Otherwise, the less immunogenic epitopes would elicit an inefficient anti-cancer T cell response, with a resulting immunological escape of tumor cells.

Epitope specificity for T cells is mediated by the T-cell receptor (TCR), which binds peptides presented in the “peptide binding groove” of class I or class II major histocompatibility complexes (MHCs, also known as human leukocyte antigen, HLA, for humans). Whole proteins are internalized and proteolyzed by APCs and the resulting short peptides are loaded onto MHCs and presented on the APC surface. Consequently, specific TCRs may bind such peptide−MHC complexes (pMHC). Peptides binding the same HLA show a signature characterized by specific aminoacid residues at the positions interacting with the MHC groove (anchor residues). In particular, HLA-A2 - restricted epitopes show the xL/IxxxxxxV/L signature, where L/I and V/L represent the anchor residues (L: Leucine; I: isoleucine; V: Valine). Indeed, their side chains are oriented toward the interior of the peptide-binding groove and mediate the anchoring of the peptide to the MHC molecule (41, 42). The residues in the other positions of the epitope point toward the TCR and mediate the specificity of the interaction between the T cell and the target pMHC complex.

However, although the peptides adopt an optimal conformation in the groove of the MHC, there is no guarantee for their immunogenicity. Therefore, in order to improve the immunogenicity of a candidate antigen it is necessary to increase both the binding and the affinity to the MHC groove and to the T cell TCR, as described below.

### 2.2 Short versus long synthetic peptides

The sequence length of peptide vaccines is important to promote a strong immunogenic response. Typically, peptides can be either short (8 – 11 amino acids) or long (11 – 30 amino acids) for presentation in MHC class I or II molecules, respectively.

Short peptides represent the nominal epitope capable of binding the class I MHC molecules. They are attractive for vaccine development as they are easy to synthesize and cheap to produce in clinical grade. However, short peptides may bind to MHC of non-professional APCs, including B and T lymphocytes, which lack the secondary signaling machinery and cannot provide the full range of costimulatory signals required for complete T cell activation. Moreover, such non-conventional antigen presentation takes place in non-inflamed lymph nodes and in the absence of a strong pro-inflammatory context (43). This would lead to a poor T cell response or immune tolerance (43). Short peptides are strictly HLA-type restricted and consequently have to perfectly match the patient’s HLA, limiting their use to a specific subset of patients (44, 45). Furthermore, they are susceptible to fast exopeptidase-mediated degradation, which dramatically reduces their half-life (46, 47).

Nevertheless, short peptides represent exactly the natural HLA ligands on tumor cells and offer the possibility for precise immunomonitoring analyses (48–51).

Alternatively, vaccines can be based on synthetic long peptides (SLPs). They cannot bind directly the MHC molecule and needs to be internalized and processed by professional APCs for presentation on both class I and class II MHC molecules. Consequently, SLPs can activate also the CD4+ T_H_ cell response, providing helper factors (interferon γ [IFN-γ], tumor necrosis factor α, IL-2), essential for sustaining the cytotoxic CD8+ T cell responses and inducing the immune-mediated tumor cell killing (52–54) (18). Lastly, they offer the possibility of including binding or recognition motifs for improved immunogenicity (55, 56).

Several early clinical trials based on SLPs have shown a good safety profile and promising results. For example, in three independent studies, SLP vaccination, based on HPV16 E6/E7 antigens, induced high levels of specific cytotoxic T lymphocytes, positively correlated with clinical benefit in premalignant disease (57, 58) and late-stage cervical cancer (59). In particular, the efficacy of SLP vaccine was potentiated when combined with immunomodulatory drugs (anti-PD-1 immune checkpoint and platinum-based chemotherapy) (59, 60).

Overall, an important condition for success of a cancer vaccine is the induction of a robust and sustained of both specific CD8+ and CD4+ T cells response as well as the increase in the CD4+:Treg ratio, to counteract the immunosuppressive TME.

### 2.3 Tumor associated antigens vs tumor specific antigens

In the last years, shared tumor antigens, also called Tumor associated antigens (TAAs), have been mostly adopted for cancer vaccine development. These include ‘self-antigens’ such as differentiation antigens (e.g., tyrosinase, gp100, MART-1), cancer/testis antigens (e.g., MAGE-A1, MAGE-A3, NY-ESO-1, and PRAME) and overexpressed antigens (7–9, 61–65). These antigenic candidates have the advantage of being shared between patients with the same tumor type, but the disadvantage of being expressed also by normal cells. Consequently, they may elicit either a limited immune response, due to self-tolerance, or a potent autoimmune response, if the self-tolerance is broken. Therefore, the appropriate balance between such two extremes needs to be reached when peptide-based vaccines are based on TAAs (65).

More recently, attention has turned to ‘neoantigens’, that comprise mutated antigens arising in tumors by non-synonymous somatic mutations, insertions/deletions (INDEL) in the coding regions, frameshifts as occurring in microsatellite-instability-high tumors (66) and human endogenous retroviruses (67). Moreover, post-translational modifications such as phosphorylation (68), glycosylation (69) or methylation contribute to express new antigens on tumor cells. These types of antigens, also named tumor specific antigens (TSAs), are distinct from the corresponding wild-type self-antigens and are not affected by immunological tolerance. Therefore, a robust specific T cell response against these antigens can be elicited (70, 71). However, TSAs are strictly private to each individual cancer patient and their identification is laborious as well as expensive (13). Moreover, they are subject to immunoediting and escape. Therefore, the neoantigen repertoire is dynamic and may evolve in the course of tumor progression, requiring a pool of neoantigens in vaccine formulation for efficacy.

## 3 Identification and design of tumor antigens for cancer vaccine

### 3.1 Isolation of human leukocyte antigen (HLA) ligands

The separation of peptides from HLA molecules is usually accomplished by acid elution using HLA-specific antibodies. This process does not destroy the structure of the peptides, which are non-covalently bound to HLA molecules. However, acid elution of HLA-presented peptides can also be performed by simply incubating living cells in medium with a low pH and harvesting HLA ligands from the supernatant (72). The major disadvantage of the latter is that the harvested supernatant contains many other materials from the cells, which may interfere with the subsequent analysis. Surgically resected tumor tissues are the most suitable source, providing the whole array of the ligands presented by the cancer cells *in vivo*. Passages *in vitro* culture are not recommended in order to avoid possible changes in the HLA ligand repertoire, due to adaptation to cell culture conditions. Whenever possible, the tumor tissue should be ideally coupled by a sample of non-tumor tissue from the same organ for selecting only tumor-specific peptides.

In addition, blood can represent a potential source of soluble HLA molecules (sHLA), which can be regularly found in both healthy and diseased individuals, deriving from death or living cells or exosomes. In cancer patients, such blood-derived sHLA molecules have also been shown to contain peptides matching those identified in cancer cells. Therefore, in case of poor availability of tumor tissue, a “liquid biopsy” could provide useful information for cancer diagnosis and monitoring as well as identification of antigenic targets useful for developing therapeutic interventions (73, 74).

### 3.2 Immunopeptidome for human leukocyte antigen ligands identification

**Figure 1:**
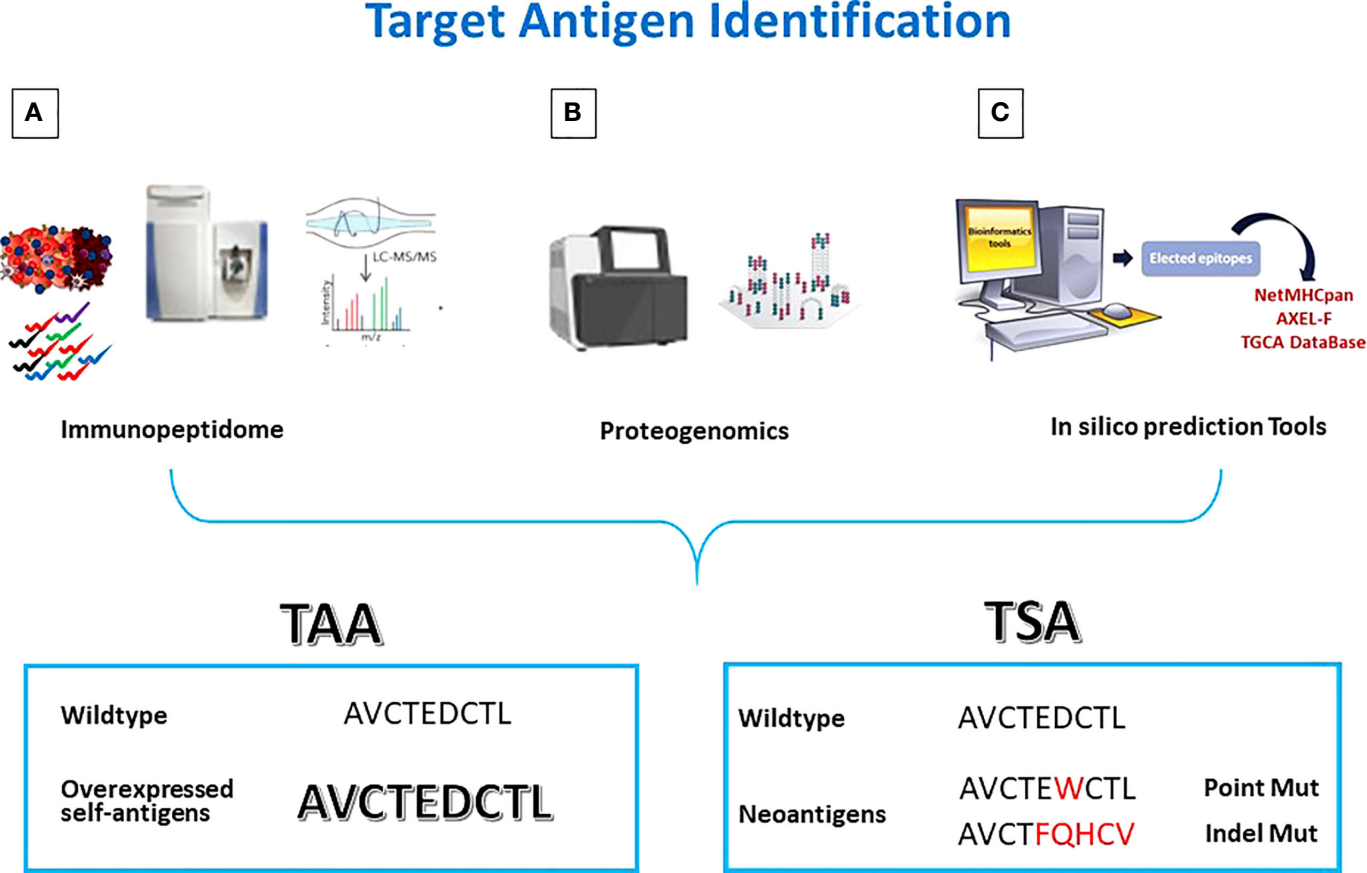
Antigen discovery strategies. Tumor antigens are discovered by **(A)** Mass Spectometry analysis of eluted peptides; **(B)** integrated multi-omics screening; **(C)** in silico prediction. TAAs are wt epitopes over-expressed in cancer cells compared to normal cells. TSAs are mutated neo-antigens.

One of the methods of choice used for this purpose is based on a comprehensive analysis of the HLA ligandome of cancer cells (also termed immunopeptidome) to pursue tumor antigen discovery (75). It allows to identify and validate the “natural” presentation of new or well-known TAAs by the majority of primary tumors compared to normal tissues (**Figure 1A**) (76, 77).

HLA molecules are precipitated from the tissue’s samples and HLA-bound peptides are eluted for MS analysis (78, 79). In particular, using high-performance liquid chromatography with very narrow long columns, peptide-HLA complex mixtures (pMHC), are separated before injection into the ionization chamber of the mass spectrometer.The ionized peptides are separated according to their mass-to-charge ratio in the mass filter, and then analyzed in the mass detector for their actual mass. Starting from the mass-to-charge ratio of the fragments it is possible to calculate the original peptide sequence. However, the probability of an incorrect assignment (false discovery rate) is high (80–82). A principal challenge here is the complexity of data analysis. Prior to applying the identified peptides for vaccine development, they should be validated by comparison with synthetic, isotope-labeled peptides on the same equipment (83).

The MS identification of peptides should be always coupled with exome and transcriptome analyses to match the genetic and phenotypic results. The analysis of the HLA ligandome allowed to uncover another “universe” of sources for HLA ligands, derived from untranslated or not completely translated sequences. Such HLA ligands, named “cryptic peptides” (84) can be derived from noncoding RNAs, introns, or short open reading frames (85). Some of such non-conventional antigens can be consider cancer-specific (86). In order to identify the latter antigens, the proteogenomic approach has been defined by combining conventional proteomics with next-generation sequencing (NGS) (**Figure 1B**). In particular, merging WGS and RNASeq analyses with Ribosome profiling (Ribo-seq), it is possible to identify an excess of novel translated unannotated open reading frames. They can be derived from alternative out-of-frame ORFs, untranslated regions (UTRs) or long non-coding RNAs. Such non-coding transcripts could generate peptide sequences that are missing in conventional protein sequence repositories. Therefore, only the combination of genomics and proteomics on the same target cancer cells can lead to the identification of novel and more immunogenic HLA ligands, expanding the repertoire of targetable epitopes for cancer immunotherapy (86–88).

### 3.3 Human leukocyte antigen ligand prediction tools

Relevant efforts have been committed to developing computational methods capable of accurately predicting peptide binding to both MHC-I and MHC-II [reviewed in (89)]. Indeed, the analysis of the HLA ligandome has shown a great limitation in sensitivity for identifying mutated neo-antigens, especially in tumors with low mutational burden (90–92). Therefore, prediction algorithms are crucial for mutated neo-antigens discovery upon transcriptomic analysis (93). A global bioinformatics consortium was established in 2016, namely Tumor Neoantigen Selection Alliance (TESLA), which includes scientists, from academia, industry, and non-profit groups. Through predictive algorithms and machine learning, the most immunogenic neoantigens can be identified for stimulating strong immune responses (94). The TESLA consortium defined standardized predictive algorithms for neoantigens discovery, allowing to create more cancer immunotherapy treatments specific to each patient (https://www.synapse.org/#!Synapse:syn21048999).

In particular, the identification of large numbers of ligands retrieved from mass spectrometry (MS) experiments and their analysis for sequence similarities has allowed the development of algorithms based on machine learning (ML) methods for prediction of the ligand affinity to a specific MHC molecule [reviewed in (95)]. Each tool is based on different prediction methods [i.e. structure-based (SB) (96), motif matrix (MM) (97, 98), sequence motif (SM), quantitative affinity matrix (QAM) (99, 100), artificial neural network (ANN) (101, 102), support vector machine (SVM) (103, 104)]. Moreover, the choice of a specific tool is based on the characteristics of the antigen (TAA vs TSA) and the MHC molecule they bind (class I or II or both). These tools are able to address the distinction between MHC binders and non-binders as well as the prediction of the binding affinity of a peptide to MHC molecules. However, these methods are not yet proficient in deterministically estimating whether a given peptide is an epitope or not (105).

Among these tools, NetMHCpan is frequently used for MHC class I molecules and NetMHCIIpan for MHC class II (106). Both algorithms cover MHC molecules from a number of different species and many HLA alleles. Over the years, they have been continually improved (107, 108), but there are still many caveats in their prediction accuracy, especially for less frequent class I HLA alleles and for class II binding peptides (109).

In very recent studies, it has been reported that peptide presentation by MHC is strongly correlated with mRNA expression of the ligand’s source protein, underlying the importance of direct correlation between the source protein abundance and MHC epitope predictions (110–115). For this reason, the epitope predictions can be further improved by considering also the abundance of peptides’ source proteins. Based on such assumption, the Antigen eXpression based Epitope Likelihood-Function (AXEL-F), together with TGCA expression data, has been shown to improve the prediction of neoantigens that are recognized by T cells (116).

## 4 Strategies for enhancing peptide immunogenicity: peptide vaccine formulation, delivery systems and immunogenic optimization

### 4.1 Formulation of peptide-based vaccines

Peptide-based vaccines represent the easiest strategy for eliciting the most focused anti-tumor T cell response, but an appropriate formulation needs to be developed. Indeed, in order to elicit a robust effector T cells response, peptide-based vaccines must deliver a sufficiently high dose to avoid the induction of T cell anergy (117). Moreover, free peptides are characterized by unfavorable pharmacokinetic properties, including short half-lives and low stability *in vivo*, which significantly reduces their immunogenicity To overcome such limitations, they need to be formulated with adjuvants (118).

Only a few adjuvants have been approved for human use to date (119), but several new molecules are under pre-clinical and early clinical development, including cytokines, saponins, mineral salts, emulsions, bacterial exotoxins, virosomes, liposomes, and immune-stimulating complexes (120). Several studies have revealed the central role of adjuvants in stimulating the innate immunity and, downstream, the antigen-specific adaptive T cell response. In particular, the ideal target for vaccine adjuvants is represented by the pattern recognition receptors (PRRs) of the innate immune system [reviewed in (121)]. Adjuvants which improve the inflammation, the delivery of antigens to DCs and their uptake and presentation to T cells, as well as increase their stability, include aluminum salts, nanoparticles, lipid vesicles, oil- and water-based formulations, and bacterial exotoxins. Among these, only alum has had limited use in cancer vaccines due to moderate activation of Th1 and CD8+ responses (122). On the contrary, oil-in-water emulsions has been shown to activate humoral and Th1 and Th2 immune responses, enhancing recruitment of granulocytes and DCs and the antigen uptake (123). Montanide, a mineral oil-based emulsion, is the most commonly used adjuvant, increasing the delivery of antigens to DCs and presentation to T cells. It has been and continues to be used in peptide cancer vaccine formulations in melanoma and renal carcinoma clinical trials (124).

Several TLR ligands have been extensively assessed as vaccine adjuvants in preclinical as well as human clinical setting, showing their ability to elicit a balanced humoral and cellular immune response [reviewed in (125)]. TLRs comprise a family of 10 receptors (126–128) each of which is capable of recognizing unique pathogen-associated molecular patterns (PAMPs). These will activate downstream genes critical to promote innate immune responses and enhance immunity against the microbe (129). TLR agonists can be incorporated into peptide-based therapeutic cancer vaccines to induce Th1 type cellular proinflammatory cytokine production, such as type I IFNs, and the efficient activation and specific expansion of antigen-specific CTL (51, 130–136). Among these, TLR4 ligands are known to enable a potent activation of APCs (137). Moreover, the TLR-9 agonist synthetic CpG oligodeoxynucleotide (CpG-ODN) was approved for the first time by the United States Food and Drug Administration for application in humans, in the Heplisav-B hepatitis B vaccine (138).

### 4.2 Delivery systems

Another key factor for the success of a peptide-based cancer vaccines is the capability of reaching the TME before degradation and being efficiently uptaken by APCs. For these aims, PLGA and Liposomes represent two examples of drug delivery systems, which have been approved by FDA after several years of experimental testing with a proven track-record in safety and biodegradability. (Food and Drug Admistration (FDA). (2021). Available at: www.fda.gov (139). Both delivery platforms can protect peptides from degradation and control their release to the spleen and lymph nodes, which contain a higher proportion of cross-presenting DCs (140, 141). In these sites, the particles are efficiently processed by DCs inducing a strong and sustained CD4+ T helper cell and cytotoxic T lymphocyte (CTL) responses (142). Indeed, their cationic charge and small size, efficiently promote a strong attraction and active uptake by DCs (143, 144). Besides PLGA and liposomes, other nanoparticles are tested as antigen delivery system, such as micelles, mesoporous silica nanoparticles (MSNs), gold nanoparticles (AuNPs) and virus nanoparticles. The delivery and the intra-cellular trafficking of nanoparticles have been optimized by selected modification of their surface (e.g., charge, structure, dimension and hydrophobicity) (145, 146) and by incorporating cell-penetrating peptides, APC-specific cellular epitopes or immune-stimulant lipid moieties (147).

Some of the nanoparticle vaccines have been evaluated in clinical trials for different cancers [reviewed in (148–150)]. Among all, liposomes have been extensively used due to their versatility, including Tecemotide— liposomes (151, 152), AS15— lipids (153), DepoVax— liposomes (154), Iscomatrix (155), Cholesteryl pullulan (CHP) nanogels (156), and virus-like nanoparticles. However, although these trials have shown induction of specific antigen immune response, none of them have resulted in a statistically significant survival benefit. Such unsatisfactory results need to be thoroughly analyzed in order to identify strategies for improvements.

Furthermore, exosomes exhibit features for application as adjuvant carriers, such as optimal size, biocompatibility, stability in systemic circulation, and target-specific delivery (157). Zitvogel et al. (158) described that tumor peptide-pulsed dendritic cells (DCs) released DEXs (exosomes derived from dendritic cells) presenting tumor antigens on the membrane, which induced *in vivo* CTL priming and consequent tumor growth suppression. Such pillar study was the first to support the development of a novel cell-free vaccine using exosomes and subsequently confirmed by other groups (159). Moreover, DEXs have been shown to stimulate cells of the innate immune system, such as natural killer cells, and the production of INF-g (160). DEXs show several advantages over DCs: higher stability, due to their lipid composition, and higher number of peptide-MHC I and –MHC II complexes on their surface (161–163). In addition, Wolfers and colleagues have reported that the Tumor Derived Exosomes (TDEs) represent a source of T-cell cross-priming, which are able to induce a CTL anti-tumor responses *in vitro* and *in vivo* (164).

Overall, these results highlight the novel use of exosomes as adjuvanting carriers for a future cancer vaccine development.

### 4.3 Peptide modification to improve the immunogenicity of antigenic targets

In order to improve the immunogenicity of tumor antigens, peptides can be modified to increase their (165)affinity and binding to the presenting MHC-I (166). Analogue peptides are designed by substituting amino acid residues in the epitope sequence to improve antigenicity and immunogenicity (167). Such modified peptides (heteroclitic peptides) have been shown to induce a more potent CD8+ T cell response, and break the immunological tolerance (86, 167–170). Nevertheless, Tor B Stuge et al., have demonstrated that although vaccination with heteroclitic peptides may induce strong T cell responses, the recognition efficiencies of the wild type peptides may significantly vary. Indeed, most of such immune responses show a poor cytotoxic effect against melanoma cells expressing the wild type epitope (165).

A different approach for improving the immunogenicity of natural TAAs is to generate heteroclitic peptides with mutations in the TCR-binding residues (42, 171). In this regard, our group has shown that heteroclitic peptides modified in the TCR-binding residues of melanoma specific Trp2 TAA can improve control of tumor growth in a mouse model (172). In particular, the modification of TCR-facing amino acids, significantly improve the recognition by PBMCs of the HPV E7 wt epitope expressed on TC1 mouse lung tumor cell lines. Consequently, heteroclitic peptides are able to elicit even stronger immune response, cross-reacting with the parental wild type peptide. CTL elicited by the heteroclitic peptides show potent lytic activity on target cells expressing the wt peptide as well as control of tumor growth *in vivo* (173).

### 4.4 Antigenic molecular mimicry between microorganisms’ antigens and TAAs

Another strategy for improving the immunogenicity of tumor antigens is to identify novel universal shared antigens able to overcome the immune tolerance and elicit an effective T cell immune response. In this respect, non-self antigens derived from microorganisms (microorganisms-derived antigens, MoAs) with high sequence and structural homology with TAAs (molecular mimicry) can be highly useful.

The probability of homology between MoAs and human antigens and an overlapping peptidome representation is high and can result in a cross-reacting CD8+T cell responses. Indeed, similar epitopes can be targeted by the same CD8+ T cell receptor (TCR), given that a single TCR is cross-reactive with at least 10 (6) different MHC bound peptides (174, 175). Therefore, two unrelated antigens are very likely recognized by the same TCRs if the structural conformation of the entire epitopes is saved, sharing the same or conserved TCR facing central residues (176). This process may drive the fate of cancer development, progression and eventually response to therapy.

Preliminary evidences for T cells cross-reacting with microbial antigens and the homologous TAAs have been reported (177–179). A more general evidence of molecular mimicry between TAAs and viral or microbiota-derived antigens has been reported by our group (176, 180–182).

Overall, if tumor cells express a TAA similar to non-self MoA, it is possible to recall memory CD8+ T cells cross-reacting with tumor antigens, able to control the tumor growth. This may at last represent a relevant selective advantage for cancer patients and may lead to a novel preventive anti-cancer vaccine strategy (183).

## 5 Combinatorial strategies

The limited efficacy of cancer vaccines is consequent to immune-evasive mechanisms developing in the TME. These can be effectively targeted by combining different strategies based on immunotherapy and conventional anticancer therapies, such as standard or metronomic chemotherapy (MCT) and radiotherapy (184–186) as well as the combination of Immune checkpoint inhibitors (ICIs). Indeed, the immunosuppressive TME leads to a limited infiltration of cancer-specific cytotoxic T lymphocytes and/or their functional anergy/exhaustion, resulting in a reduced efficacy of cancer vaccines.

### 5.1 Chemotherapy and vaccines

Numerous preclinical and clinical studies have been conducted in recent years to evaluate the efficacy of combinatorial strategies in enhancing the efficacy of the cytotoxic immune response induced by cancer vaccines.

Standard and metronomic chemotherapy, such as oxaliplatin and doxorubicin, induce positive immunomodulatory effects in the TME and can enhance the antitumor immune responses elicited by cancer vaccines, by inhibiting the immunosuppressive cells (i.e., Tregs and MDSCs) and increasing interferon gamma (IFN-γ) secreting CD8+ T cells. Moreover, it induces immunogenic cell death (ICD) in cancer cells, with the release of danger signals able to polarize dendritic cells (DCs) and activate an antitumor T helper 1 (Th1) responses. Consequently, the immunogenicity of tumor cells is modulated, becoming more susceptible to T-cell-mediated lysis (187–189). Additionally, chemotherapy increases the tumor mutational burden leading to the release of an increased load of neoantigens into the TME (190–195). Several pre-clinical studies, including from our group, have confirmed and demonstrated the immunomodulatory effects of standard and metronomic chemotherapy in potentiating the efficacy of a cancer vaccine (38, 196, 197). However, when the same protocols have been transferred in the clinical setting, partial results and non-lasting effect have been observed (49, 198, 199).The possible reasons for such failure are: (1) the choice of chemotherapy drugs, their correct dose, schedule and combination; and (2) the selection of patients’ cohort. Therefore, additional work is required to fully decode the pharmacological and immunological interactions between peptide-based anticancer vaccines and chemotherapy.

### 5.2 Radiotherapy and vaccines

Similar to chemotherapy, also radiotherapy (RT) can significantly increase the efficacy of vaccines. Indeed, it induces significant changes in the TME, with the release of both antigens from tumor cells and pro-inflammatory mediators. Overall, these effects increase tumor immune infiltration and trigger the innate as well as adaptive immune system (200, 201). The latter effect drives the systemic antitumor immunity which underlies the so-called “abscopal effect,” consisting in the capacity to eradicate distant metastasis (202–206). Overall, immunologically ‘cold’ tumors are converted into ‘hot’ tumors. Moreover, RT is considered an “*in situ* vaccination”, because induces DNA damage, altered gene transcription and tumor neoantigen expression. Several studies have shown an increased anticancer immune responses when RT is administered both before and after a tumor-specific therapeutic vaccine. In particular, in human papillomavirus (HPV)- driven cancer models it has been shown that the combination of radiotherapy with vaccination strategies based on HPV E7 induces more efficient, DC maturation and peptide- specific T cell responses (207, 208).

### 5.3 Immune checkpoint inhibitors and vaccines

Immune checkpoint inhibitors (ICIs) are able to unleash the T cell response in the TME (209) and can reprogram the activation of T cells functionally exhausted by the chronic exposure to an antigen (117, 210, 211). Unleashing of antitumor T cells by ICIs represents the optimal strategy to complement anticancer vaccines. Indeed, anti-tumor T cells induced by the cancer vaccines would be more effective in a favorable TME generated by the ICIs. In particular, the immunomodulatory effect of ICI has been evaluated in a preclinical setting, showing the induction of CTL activation and the inhibition of immunosuppressive cells (MDSC and Tregs) (ref). In addition, several clinical trials have assessed cancer vaccines combined with a checkpoint inhibitor, such as anti-CTLA-4, PD-1, or PD-L1 (https://clinicaltrials.gov/ct2/results?term5cancer1vaccines+%261immune+checkpoint+inhibitors&Search=Apply&recrs=a&age_v=&gndr=&type=&rslt).

In details, the most significant results were observed in two clinical trials with T-VEC and sipuleucel-T vaccines, where the combination with ICIs induced higher antitumor activity and objective response rate (ORR) compared to the ICIs alone (212, 213). The combination of SLP vaccine ISA101 and anti-PD-1 immune checkpoint nivolumab was shown to be well tolerated in 24 patients with HPV-16–positive cancer. Moreover, the efficacy of combinatorial strategy appeared superior to that of nivolumab monotherapy (59). Other clinical trials have assessed the combination of ICI and cancer vaccines with contrasting efficacy data. Additional studies are required to identify the most appropriate clinical settings for optimal efficacy.

### 5.4 IDO Inhibitors

Indoleamine 2,3-dioxygenase (IDO) 1, an enzyme catabolizing tryptophan to kynurenine, generates immunosuppression in TME and his over-expression is correlated with tumor progression (214). In particular, several preclinical studies have shown that over expression of IDO1 correlates with an increase of Tregs and MDSC cells, and upregulation of PD-1 in cytotoxic T cells (215). In different tumor settings (i.e. colon cancer, cervical cancer, melanoma) high level of IDO1 was associated with poorer outcomes (216, 217). For these reasons, therapeutic strategies based on IDO1 inhibitors combined with a cancer vaccine, could be a promising approach. Indeed, small-molecule inhibitors of IDO 1 as well as peptide vaccines derived from IDO were tested in early phase clinical trials in combination with ICIs, demonstrating the safety and promising immune and clinical responses (NCT05155254) (218, 219).

## 6 Conclusions and future prospective

**Figure 2:**
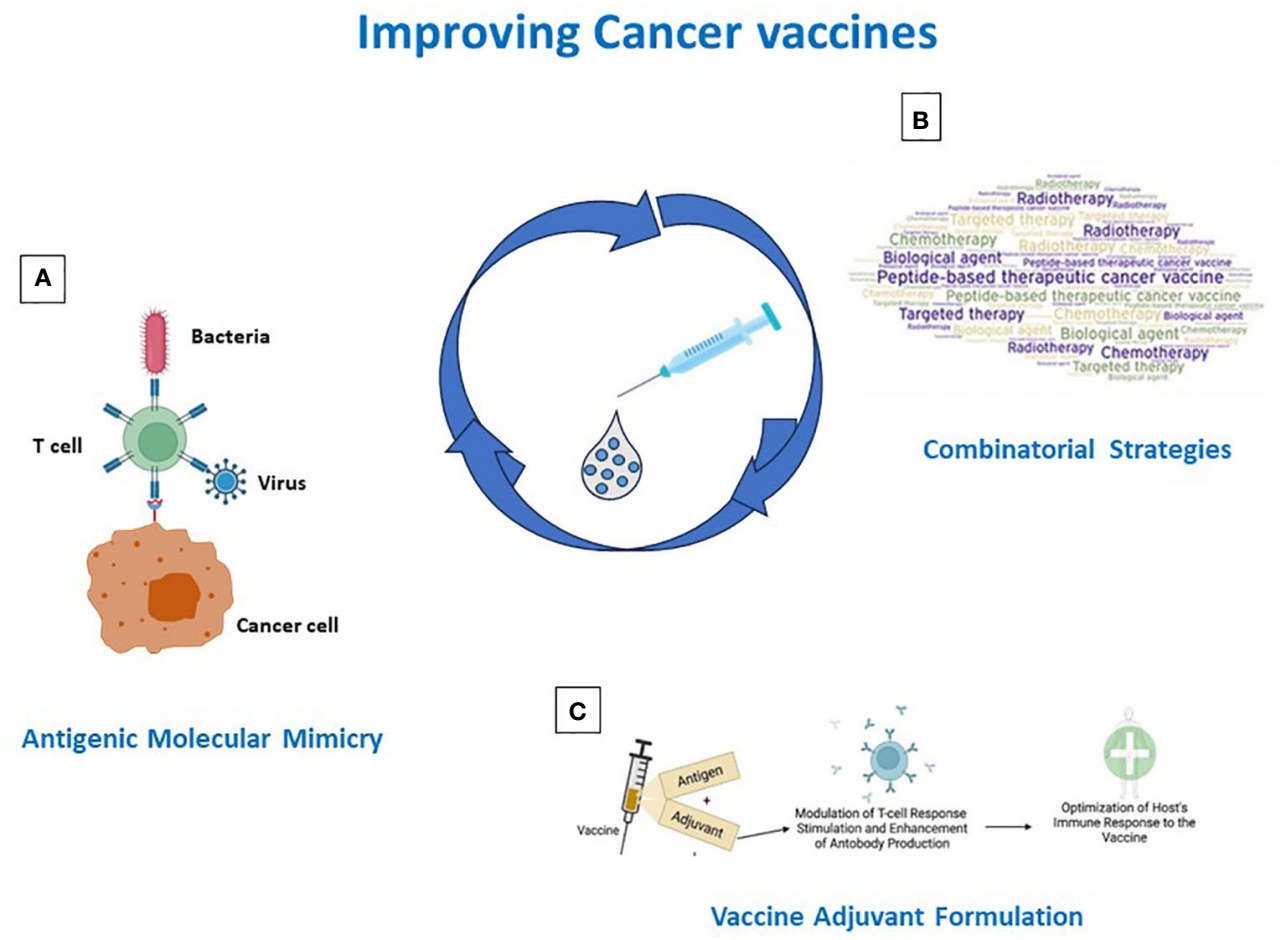
Strategies for improving cancer vaccine immunogenicity. Immunogenicity can be improved by **(A)** identification/generation of TAA-like non-self antigens; **(B)** design of combinatorial strategies including vaccines and standard and IO therapies; **(C)** antigen formulation in Th-1 driving adjuvants.

Over the years, various efforts have been made in developing cancer peptide vaccines, but their effectiveness has been limited. Several reasons are responsible for such failures, including the selection of poor immunogenic target antigens and the strong immunosuppressive TME. Strategies to address and overcome each of such aspects are currently pursued in the field. Derivatives of TAAs are designed (e.g. heteroclitic peptides, homologous non-self antigens) and TSAs are selected to improve the antigen immunogenicity. The latter include both mutation-derived as well as unconventional antigens, which, however, represent a great technological challenge and are currently of limited feasibility on a large scale. Likewise, combination treatments (e.g. vaccines with chemotherapy or radiotherapy or ICIs) are evaluated to counterbalance the immunosuppressive TME (**Figure 2**). In addition, inefficient delivery systems, patients’ selection and loss of MHC class-I may contribute to failures, but these aspects are beyond the scope of the present review article (144, 220). Consequently, several issues remain to be solved.

Indeed, although a significant number of early stage clinical trials,based on peptide-based vaccines, are currently recruiting around the World (nr. 64 as of November 2022), a single trial is beyond phase II (NCT03284866) (https://clinicaltrials.gov/ct2/results?term=peptide+cancer+vaccine&recrs=a&age_v=&gndr=&type=&rslt=&Search=Apply). This confirms the limited efficacy of peptide-based cancer vaccines.

Therefore, further studies are necessary to clarify how to identify new efficient strategies able to give to peptide-based cancer vaccines a new horizon.

## Author contributions

MT and LB conceived and drafted the review. All authors contributed to the article and approved the submitted version.
