# Corrigendum: Peptide-based vaccine for cancer therapies

**DOI:** 10.3389/fimmu.2023.1324894

**Published:** 2023-10-26

**Authors:** Luigi Buonaguro, Maria Tagliamonte

**Affiliations:** Innovative Immunological Models Unit, Istituto Nazionale Tumori - IRCCS - “Fond G. Pascale”, Naples, Italy

**Keywords:** peptides, TAA, TME, molecular mimicry, immunopeptidome, combinatorial strategies, adjuvant, MHC

In the published article, there was no Funding statement. The correct Funding statement appears below.

**FUNDING**

The study was funded by the Italian Ministry of Health through Institutional “Ricerca Corrente” (LB and MT) L2/3 project to LB “Tumor antigen discovery for innovative cancer immunotherapies in HCC: from benchside to bedside (HepAnt)”. L2/13 project to MT “Peptide optimization to improve antigen presentation and anti-tumor response for sarcomas”.

The authors apologize for this error and state that this does not change the scientific conclusions of the article in any way. The original article has been updated.

